# RNA-Seq Identified Putative Genes Conferring Photosynthesis and Root Development of Melon under Salt Stress

**DOI:** 10.3390/genes14091728

**Published:** 2023-08-29

**Authors:** Tai Liu, Sikandar Amanullah, Huichun Xu, Peng Gao, Zhiqiang Du, Xixi Hu, Mo Han, Ye Che, Ling Zhang, Guochao Qi, Di Wang

**Affiliations:** 1 Daqing Branch of Heilongjiang Academy of Agricultural Sciences, Daqing 163711, China; liutai1997@sina.com (T.L.); xhc836060@sina.com (H.X.); duzhiqiang2023@sina.com (Z.D.); huxixi116@sina.com (X.H.); dqnkyhm@sina.com (M.H.); cheye12345@sina.com (Y.C.); zhang8374ling@sina.com (L.Z.); chao129129@sina.com (G.Q.); 2 Key Laboratory of Biology and Genetic Improvement of Horticulture Crops (Northeast Region), Ministry of Agriculture and Rural Affairs, Northeast Agricultural University, Harbin 150030, China; sikandaraman@yahoo.com (S.A.); gaopeng_neau@163.com (P.G.); 3 College of Horticulture and Landscape Architecture, Northeast Agricultural University, Harbin 150030, China

**Keywords:** melon, salt stress, photosynthesis, membrane-lipid metabolism, growth inhibition

## Abstract

Melon is an important fruit crop of the Cucurbitaceae family that is being cultivated over a large area in China. Unfortunately, salt stress has crucial effects on crop plants and damages photosynthesis, membranal lipid components, and hormonal metabolism, which leads to metabolic imbalance and retarded growth. Herein, we performed RNA-seq analysis and a physiological parameter evaluation to assess the salt-induced stress impact on photosynthesis and root development activity in melon. The endogenous quantification analysis showed that the significant oxidative damage in the membranal system resulted in an increased ratio of non-bilayer/bilayer lipid (MGDG/DGDG), suggesting severe irregular stability in the photosynthetic membrane. Meanwhile, root development was slowed down by a superoxidized membrane system, and downregulated genes showed significant contributions to cell wall biosynthesis and IAA metabolism. The comparative transcriptomic analysis also exhibited that major DEGs were more common in the intrinsic membrane component, photosynthesis, and metabolism. These are all processes that are usually involved in negative responses. Further, the WGCN analysis revealed the involvement of two main network modules: the thylakoid membrane and proteins related to photosystem II. The qRT-PCR analysis exhibited that two key genes (*MELO3C006053.2* and *MELO3C023596.2*) had significant variations in expression profiling at different time intervals of salt stress treatments (0, 6, 12, 24, and 48 h), which were also consistent with the RNA-seq results, denoting the significant accuracy of molecular dataset analysis. In summary, we performed an extensive molecular and metabolic investigation to check the salt-stress-induced physiological changes in melon and proposed that the PSII reaction centre may likely be the primary stress target.

## 1. Introduction

In China, melon fruit crops are mainly cultivated in open fields during the normal season and in greenhouse conditions during the off-season. The planting area, average yield, and other commercial benefits of melon are increasing day by day, but the various biotic and abiotic factors are also becoming very serious and limiting the sustainable development of the melon industry. With advanced cultivation technologies, the accumulation of soil salinization is also increasing, which has had a serious effect on the yield and quality of melons.

Salinity is one of the most devastating environmental stresses limiting crop yield due to salt-sensitive mechanisms [1], and the area of global land affected by high concentrations of salts is still expected to increase due to damaging agricultural practices and climate change. Soil salinity can alter photosynthesis, membrane–lipid metabolic processes, and hormonal metabolism in plants, thereby causing metabolic disorder, organelle dysfunction, and ion toxicity [1,2]. The effects of salt-induced stress on the endogenous physiological activities of plants show that root vitality is decreased and the plant bears a gradual reduction in photosynthesis rate and leaf area, which ultimately results in leaf wilting, yellowing, and stunted growth [3].

Some plants can react to salinity-induced stress by triggering their internal photosynthesis activity. For instance, the gene encoding photosystem I (PSI) reaction center subunit IV (PsaE) was enhanced in the wild haplotype of *Oryza coarctata* [4]. The accumulation of excessive sodium (Na^+^) can be prevented in chloroplasts via upregulation of the circular-type electron flow across PSI in soybean crop plants [5]. Based on this evidence, plants exhibit different responses towards photosynthesis inhibition disturbed by salt-induced stress, which also reveals the importance of dissecting the physiological mechanisms regulating plant photosynthesis and growth activity.

The cellular membrane serves as a protective blockade between plants and their external stimuli due to its intrinsic components and particular arrangement [6]. There are three major kinds of plant-membrane-associated lipids, and the most prevalent and primary structural constituent is glycerolipids [7,8]. Glycerolipids could associate with two types of fatty acids in the glycerol structure and merge with phospholipids or glycolipid molecules [9]. The plastid bilayer membrane is composed of a combination of monogalactosediacyl glycerol (MGDG), digalactosyldiacylglycerol (DGDG), sulfoquinovosyldiacylglycerol (SQDG), and phosphatidylglycerol (PG) [10]; however, MGDG and DGDG are known as primary lipids found in green tissues, which modulate the internal chloroplast structure and photosynthetic properties [11]. Lipid remodelling represents the alteration of head groups between various membrane lipids [12], which occurs frequently in the presence of different environmental stimuli. For instance, when the levels of DGDG increase under cold stress, the MGDG/DGDG ratio declines to enhance cell membranal stability [13,14]. Such stress-induced variations in membrane-associated lipid content in plants will help sustain membrane stability and integrity [15,16].

The evolution of crops has allowed different plants to develop multiple mechanisms to protect against salt stress. The major modulatory pathways are separated into two categories: physiological and molecular regulation. The physiological regulation can alter plant morphology by regulating hormone secretion and distribution to evade salt stress. For instance, auxin significantly influences the architecture and development of the root system, while the endogenous activity of auxin in roots could be critically altered through salt stress effects [17]. Meanwhile, physiological regulation can also modulate the enzymatic functions of antioxidants; for example, peroxidase (POD) and superoxide dismutase (SOD) regulate the excessive reactive oxygen species (ROS) and alleviate the superoxidized membrane system induced by stress [18]. Therefore, antioxidant activity, malondialdehyde (MDA) content, and conductivity (REC) can be jointly used to evaluate the degree of cell damage [19]. On the other hand, molecular regulation is far more complicated than physiological- and biochemical-based regulations [20], and there has been a significant upsurge in the exploration of molecular mechanisms of the transcription factors or gene families that modulate the various functional genes and proteins to promote salt tolerance in crop plants through cell wall remodelling enzymes and hormone synthesis genes [21,22].

In the modern era, fast-paced next-generation sequencing has facilitated the use of a novel transcriptomic analysis technology called RNA sequencing (RNA-Seq). The strategy of this technology enables the accurate determination of mRNA expressions compared with traditional sequencing methods and clearly identifies the pathways that respond to certain stimuli [23]. RNA-Seq is commonly used for the exploration of genome-wide evolution, stress response mechanisms in multiple organisms [24], and stress-induced regulatory genes in model and non-model crops, e.g., *Arabidopsis thaliana*, rice, barley, and maize, to better elucidate the underlying mechanism of plant stress resistance [25]. Recently, many researchers have used Illumina sequencing to assess the differentially expressed gene (DEG) profiles responsible for regulating the various crop traits under saline–alkali stress. The elucidation of these changes at the transcriptomic level facilitates an in-depth understanding of key biological and physiological mechanisms. It was revealed that DEGs are specifically involved in different time periods under salt-induced stress conditions, e.g., growth and development, nutrient transport and metabolism, plant defense, antioxidants, and transcriptional regulation, indicating that these types of biological pathways are critical for regulating plant growth and development [26,27]. Further, the weighted gene co-expression network (WGCN) analysis method has been frequently used for investigating the associations between genes and associated phenotypes to analyse high-throughput sequencing data and similarly facilitates the detection of essential genes accountable for traits of interest in crop plants [28,29].

Melon (*Cucumis melo* L.) is known as a significant fruit crop in the Cucurbitaceae family, but few of the botanical cultivars are severely resistant to salt stress. In this experiment, we selected the salt-susceptible variety of melon for identifying the stress-induced impact on the photosynthetic function and the change in expression profiles of root development-related genes, coupled with phenotypic and physiological observations. We found important synergistic network responses during the development of melon plant, which further assists in understanding the key genes and metabolic pathways involved in coping with salt-induced stress.

## 2. Materials and Methods

### 2.1. Experimental Material and Salt Stress

The seeds of melon variety (Longqing) were provided by the Heilongjiang Academy of Agricultural Sciences (HAAS), chosen as experiment material, and planted in the Horticulture Laboratory of HAAS. The experiment was conducted in a completely randomized design (CRD) using three replications of each salt-induced treatment. For the development of seedlings and salt stress treatments, melon seeds were first put in a muslin bag to germinate in dark light at 30 °C, and about 3-leaf stage seedlings were shifted into a hydroponic chamber containing Hoagland’s nutrient solution and grown at 25–22 °C with a light/dark cycle (16 h/8 h). The seedlings were acclimated in Hoagland solution for a total of 48 h and then treated with sodium chloride (NaCl) solution concentration (250 mmol·L^−1^). The Hoagland solution treatment without any salt stress was used as a mock control (CK). All the developed seedlings were visually observed on a regular basis, and samples were collected at different time intervals after the salt-stress-induced treatments (0, 6, 12, 24, and 48 h). The endogenous physiological and metabolic activities of melon seedlings were checked, RNA-seq data were analysed, candidate DEGs were identified, and qRT-PCR analysis was performed for practical validation, respectively.

### 2.2. Evaluation of Metabolic Activity of Roots and Chlorophyll Contents of Leaves

The samples from developed seedling roots were collected in three biological replications from the salt-stress-induced treatment interval (0 to 48 h), and the metabolic activity of root was determined. A total of 0.30 g of root samples was used for measuring the root activity by performing the 2,3,5-triphenyltetrazolium chloride (TTC) protocol [30].

Then, the chlorophyll contents of leaf samples were checked using the extraction method of ethanol–acetone. For this purpose, a total of 0.30 g of leaf samples with fresh weight (FW) was cut from the main vein of leaves attached at the identical node by using a small, sharp cutting razor, and the collected samples were dipped in a 10 mL solution of ethanol–acetone (1:1) for about a total of 24 h. The absorbance rate of extract was measured at 440 nm, 663 nm, and 645 nm using the NanoDrop™ One/One C Microvolume UV-Vis Spectrophotometer (Thermo Fisher Scientific, Delaware, DE, USA) with its user-defined custom method [31,32], and total chlorophyll contents were quantified based on the following equation:

Total chlorophyll (mg·g^−1^ FW) ¼ = (8.04 × A_663_ + 20.29 × A_645_) × V/W × 1000 (1)

where A is absorbance rate, W is weight, and V is volume, individually.

### 2.3. Identification of Antioxidant Enzyme Activity and Cell Membrane Permeability

The relative electrical conductivity (REC) of leaves was checked in percentage (%) using the earlier reported protocol of Liu et al. [30], with the following formula:

REC (%) = EC1/EC2 × 100%(2)

where EC1 and EC2 are the initial and final EC, respectively.

The lipid peroxidation and MDA contents were determined from leaf homogenates using the 2-thiobarbituric acid (TBA) protocol [30]. For the SOD determination, a total of 0.50 g of leaves was homogenized in 10 mL of 50 mM phosphate-buffered saline (PBS, pH 7.80) with 1% polyvinylpolypyrrolidone (PVPP), and centrifugation was carried out at 15,000× *g* for 20 min at 4 °C. SOD was determined using a UV-spectrophotometer (Beckman DU520) with colorimetric quantification at 560 nm, and presented as a unit per gram of fresh weight per minute [33].

For the POD determination, the reaction system included 0.90 mL of the viscous phenolic compound guaiacol (20 mmol.L^−1^), 0.10 mL of enzyme solution, 1 mL of H_2_O_2_ (30 mmol/L), and 1 mL of 50 mmol.L^−1^ phosphate buffer with pH 7.0. The POD activity was similarly quantified through a UV-Vis spectrophotometer (Beckman DU520) at 470 nm and calculated by variations in the absorbance rate of 240 nm at 30 s duration after the initiation of the first bio-reaction. POD was displayed as a unit per gram of fresh weight per minute [33].

The lipids were extracted from melon seedling leaves using Narayanan’s method with slight modifications [34]. In brief, a total of 3 mL solution of isopropanol (with 0.01% butylated hydroxytoluene (BHT)) was put into a tube and initially heated to 75 °C; freshly sampled leaves were rapidly added and then finally heated for 15 min. Then, a total of 1.50 mL of CHCl_3_ and 0.60 mL ddH_2_O were added up, and the prepared solution was allowed to shake at ambient heat for 1 h. The supernatant extract was shifted to a fresh empty tube, 4 mL of CHCl_3_:CH_3_OH (2:1) solution (having 0.01% BHT) was further added, and the mixture was shaken for 30 min until the leaves turned white. Next, 1 mL of potassium chloride (KCl) solution of 1 mol·L^−1^ was added to the extraction mixture for supernatant formation. The extracted lipid was rinsed with 2 mL of ddH_2_O and allowed to evaporate in a nitrogen blowing apparatus, again dissolved in 1 mL of CHCl_3_ solution, shifted into a 2 mL bottle, and allowed to evaporate again using a nitrogen stream. Finally, the lipid content and its composition were quantified with the electrospray ionization (ESI) method using mass spectrometry (MS).

The relevant phenotypic and molecular data were recorded in numerical values and analyzed using statistical software (SPSS, v26.0). The significance results were evaluated separately using Duncan’s test at different significance levels, respectively.

### 2.4. Analysis of Transmission Electron Microscopy (TEM)

The cytological observations of developed seedling leaves were carried out using a transmission electron microscopy analysis. In brief, the fully expanded leaf samples were collected from melon seedlings grown under the salt-induced treatments at different time intervals. The fresh leaves were quickly cut into strips of 2 mm width, and the strips were fixed in a 2.5% solution (*v*/*v*) of glutaraldehyde and further fixed with 1% osmium tetroxide. The tissues were dehydrated through a series of acetone solutions, embedded in EMBed-812 prior to thin sectioning on Ultracut UCT (Leica), and viewed using a transmission electron microscope (H-7500, Hitachi, Tokyo, Japan).

### 2.5. Analysis of Transcriptomic Sequencing

Firstly, total RNA was extracted from the whole-plant samples collected at 0, 6, 12, 24, and 48 h using the TRIzol method (Invitrogen, Carlsbad, CA, USA), and quantified using the Agilent 2100 Bioanalyzer (Agilent Technologies, Santa Clara, CA, USA). Then, the integrity of extracted RNA was assessed with an upgraded NanoDrop^TM^ spectrophotometer (Thermo Fisher Scientific, Delaware, DE, USA).

For the transcriptomic sequencing, a total of 1.50 μg RNA of each sample was used for the preparation of sequencing libraries by following the recommended protocol of NEBNext^®^ Ultra™ RNA Library Prep Kit for Illumina^®^ (Nebraska, NE, USA). Initially, messenger RNA was typically isolated using Poly T oligo-attached magnetic beads (Novogene). RNA fragmentation was completed using divalent cations at a raised temperature in an optimized NEBNext RNA First Strand Synthesis Reaction Buffer (5X). First-strand complementary DNA (cDNA) was synthesized using an M-MuLV Reverse Transcriptase (RNase H) and random hexamer primer. The second-strand cDNA synthesis was subsequently carried out using DNA Polymerase I and RNase H, and the remaining extensions were transformed into blunt ends through the polymerase activities. After adenylylation of the 3′ ends of DNA fragments, NEBNext adaptors with a hairpin loop structure were ligated for preparation of hybridization.

In the end, PCR products were filtered, and the quality of the prepared library was checked using the automated Agilent 2100 Bioanalyzer. The prepared library was sequenced using the sequencing protocol of the Illumina NovaSeq 6000 Sequencing Platform of the Beijing Allwegene Technology Company Limited (Beijing, China), and 150 bp paired-end reads were generated and transcriptomic sequencing was carried out. The raw sequence reads’ RNA-seq data were uploaded to the online Sequence Read Archive (SRA) database (Accession: PRJNA987131, containing 15 biosamples) of the National Center for Biotechnology Information (NCBI).

### 2.6. DEG Identification and Analysis

To validate the transcription levels of DEGs, fragments per kilobase of transcripts per million mapped (FPKM) reads were used to count the relative gene expression using StringTie software. The DEGs of Longqing variety at different time intervals were grouped based on the clustering method of the STEM (short time-series expression miner) using the DESeq software analysis of gene expression differences, and the following thresholds were set: qValue < 0.05 and |FoldChange| > 2. Finally, the detailed high-level systematic functions and integration of DEGs were explored by gene ontology (GO) and Kyoto Encyclopedia of Genes and Genomes (KEGG) functional enrichment analysis using TBtools software and R language tool (v-4.3.1) [35,36]. We also used the KOBAS software and computed the enrichment of DEGs in KEGG pathways.

### 2.7. qRT-PCR Analysis

The gene primers were designed using Primer Premier (v6.0) software, and relative gene expression levels and transcriptomic data were validated using qRT-PCR analysis, respectively. The fold changes in the relative expression levels were tested using the 2^−ΔΔCT^ (−delta delta CT) protocol, and *Actin* was used as the reference gene. In brief, a total of 10 μL of reaction mixture was prepared that contained 1 μL of cDNA, 0.2 μL of each primer (10 μM), 5 μL of 2 × SYBR Green PCR Master Mixture, and 3.60 μL of distilled water. In total, three biological replicates of sampled tissue and at least three technical replicates of each biological replicate were used for subsequent gene expression analysis. The PCR reactions were carried out on an Applied Biosystems (ABI, 7300) Fast Real-time PCR System using the following thermocycle conditions: 95 °C for 10 min, 40 cycles at 95 °C for 15 s, 60 °C for 15 s, and 72 °C for 15 s, respectively.

### 2.8. WGCN and Pathway Analysis

The genes were filtered, gene expression trends were recorded, and gene co-expression modules were developed using a weighted gene co-expression network analysis (WGCNA, v1.47) [37]. The correlation coefficients of expression of other genes were counted to explore the appropriate threshold level for developing the gene networks using a scale-free topology model [38]. After that, module samples were subjected for an analysis of correlation coefficient values and biologically candidate modules. GO enrichment pathway analyses were carried out to evaluate the essential biological function of genes in each module using a q-value of <0.05 as a threshold.

MapMan figures were generated by operating the Mercator tool (http://mapman.gabipd.org/web/guest/mercator (accessed on 1 April 2023)) with default parameters for assigning the MapMan bins to melon transcripts. The Log2 fold changes as obtained from DESeq output were used as MapMan input to represent expression changes. The Bioconductor package Pathview version (v1.6.0) was used to generate the KEGG pathway picture, incorporating colour-coded expression values. The KEGG database integrates genomic information with higher-order functional information by collecting manually drawn pathway maps representing existing information on cellular processes and standardized gene annotations. The pathview parameters were set as default ones, and the limit parameter was set as: limit = list (gene = 5, cpd = 1). As per the default settings of Pathview, log2-fc values for boxes representing more than one gene were summed up.

## 3. Results

### 3.1. Phenotypic and Cytological Observations of Melon Seedlings

**Figure 1:**
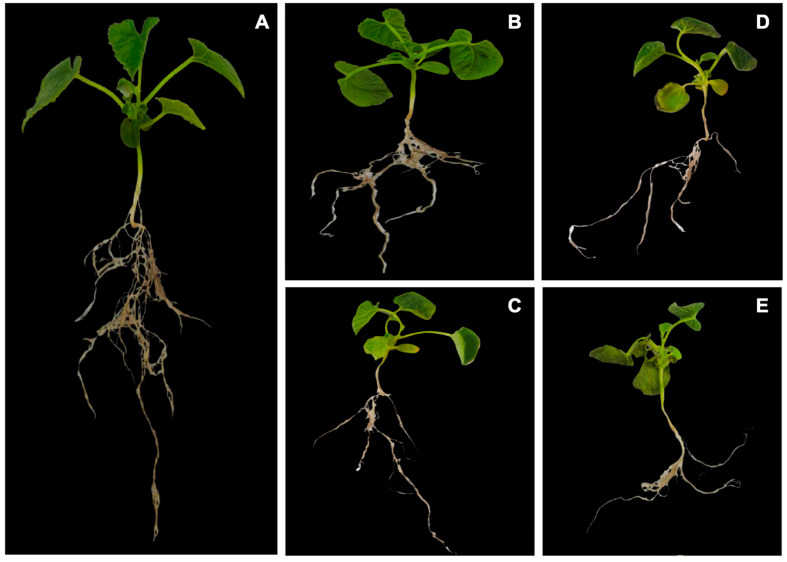
The observed phenotypes of melon seedlings grown under salt stress treatments at (**A**) 0 h (CK) interval; (**B**) 6 h interval; (**C**) 12 h interval; (**D**) 24 h interval; (**E**) 48 h interval.

To explore the salt-induced stress changes, we evaluated the physiological parameters associated with root development and metabolism. The obtained results exhibited that the salt stress depicted a significant effect on developed leaves and roots; however, the shoot tissue was highly susceptible to salinity as compared to the root tissue, and the lateral root development was more severely suppressed under salt stress relative to primary root (Figure 1).

**Figure 2:**
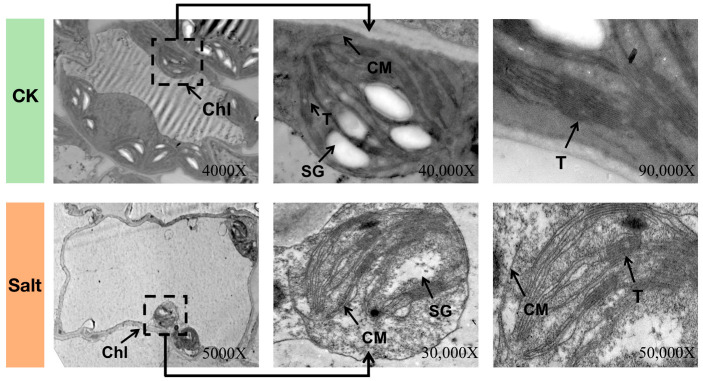
An internal ultrastructure of organelles in melon seedling leaves was observed under salt stress (48 h time interval) and control (CK, no salt) treatments. Chl, chloroplast; CM, chloroplast membrane; T, thylakoid; SG, starch granule.

The cytological observations of the internal ultrastructure of leaves’ organelles clearly exhibited that the internal chloroplasts had a typical configuration with an appropriate structure of thylakoid in the seedlings grown under CK treatment (without salt stress) at 0 h. However, the chloroplasts became shrivelled and displayed a spindle shape, the thylakoid arrangement became uneven and unclear, and the starch granule quantity decreased and their integrity was slowly degraded after 48 h of salt stress treatment (Figure 2).

The internal ultrastructure of the organelles of the developed seedling leaves was checked under salt stress treatment using transmission electron microscopy (Figure 2). The chloroplasts clearly exhibited a typical configuration with an appropriate structure of thylakoid in the seedlings grown under CK treatment (without salt stress) at 0 h. However, the chloroplasts became shrivelled and displayed a spindle shape, the thylakoid arrangement became uneven and unclear, and the starch granule quantity decreased and their integrity was slowly degraded after 48 h of salt stress treatment.

### 3.2. Physiological Variations in Leaves of Melon Seedling

**Figure 3:**
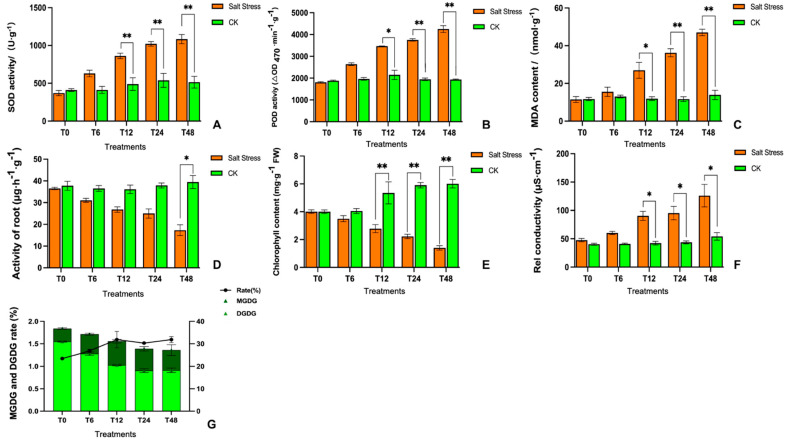
Physiological analysis of melon seedlings grown under salt-induced stress treatments at different time intervals. (**A**) SOD activity, (**B**) POD activity, (**C**) MDA content, (**D**) root activity, (**E**) chlorophyll content, (**F**) relative conductivity, and (**G**) enzyme activity rate indicate the changes under salt treatment intervals at both significance levels (** *p* < 0.01 and * *p* < 0.05), respectively.

Based on the physiological indices, we observed a marked upsurge in SOD and POD activities (Figure 3A,B) that damaged the electron transport networks due to the synergistic effect of imbalanced cellular homeostasis. The MDA contents in the leaves were noticeably higher by 40% as compared to the CK (control) and after 48 h of salt-induced treatment (Figure 3C). The root metabolic activity also reduced drastically by 56% at the 48 h interval (Figure 3D), and this potentially contributed to reduced leaf development. However, the lateral roots became dehydrated after 6 h of stress treatment and rapidly lost their vitality (Figure 1).

Further, the dramatic loss of chlorophyll content was also consistent with the observed changes in the internal ultrastructure of the chloroplasts (Figure 2 and Figure 3E), which similarly indicated cell membrane damage confirmed by the enhanced REC values (Figure 3F). The molar percentage galactolipids MGDG and DGDG dominated the overall leaf glycerolipid contents, simultaneously taking up over 60% of the glycerolipid contents, with MGDG contributing to 37% and DGDG approximately 30% of the entire glycerolipid concentration. The MGDG content was significantly reduced by 20.30% with the prolongation of treatment under salt stress, and the DGDG molar percentage decreased to 21.89% at 48 h of treatment (Figure 3G).

### 3.3. Overview of Transcriptomic Data

A total of 13,092 DEGs were sorted, and their corresponding distributions can be seen in a VENN diagram (Figure S1A). As illustrated, a total of 565 DEGs were localized in four sample combinations; however, the DEGs (8964) of T48 vs. T0 treatments were more abundant than the DEGs (2854) of T6 vs. T0 treatments, which indicated that a majority of the DEGs were late-response genes (Figure S1B). The hierarchical DEG clustering based (Figure S1C) revealed that there were higher counts of downregulated genes as compared to the upregulated genes under salt stress conditions.

Then, all DEGs were multi-aligned with the GO database and better physiological functions were elucidated. A total of 30 DEGs with the leading functional groups were annotated; among them, the oxidation–reduction process, transmembrane transport, carbohydrate metabolic activity, intrinsic component of membrane, and oxidoreductase activity were heavily enriched during the early phase of stress (Figure S2A,B). These results suggest that salt stress effectively modulated the numerous physiological and biochemical processes that were related to metabolic activities and REDOX regulation. However, most of the DEGs were annotated in the membranal and cellular component between 24 and 48 h, indicating that with the depth of stress, the metabolism of the membrane system gradually occupied the dominant position of regulatory stress (Figure S2C,D).

Further, DEGs were plotted to the KEGG reference canonical networks, and the biochemical, metabolic and signal transduction pathways under salt stress were detected. The results signified that metabolism regulation was the dominant pathway during salt stress, followed by genetic, cellular, and environmental information processing. The candidate DEGs were noticed more in the process of fatty acid metabolism and the degradation of valine, leucine, and isoleucine, plant hormone signal transduction, as well as the peroxisomal pathway between 6 and 48 h of salt stress treatment (Figure S3A–D). In contrast, the downregulated DEGs were more in the process of carbon fixation within the photosynthetic organisms, phenylpropanoid biosynthesis, carbon metabolism, photosynthesis, as well as glycine, serine, and threonine metabolisms (Figure S3E–H). Altogether, this evidence suggests that photosynthesis, fatty acid metabolism, and carbon metabolism were the major pathways affected by salt stress in melon.

### 3.4. DEGs Associated with Key Biological Processes

**Figure 4:**
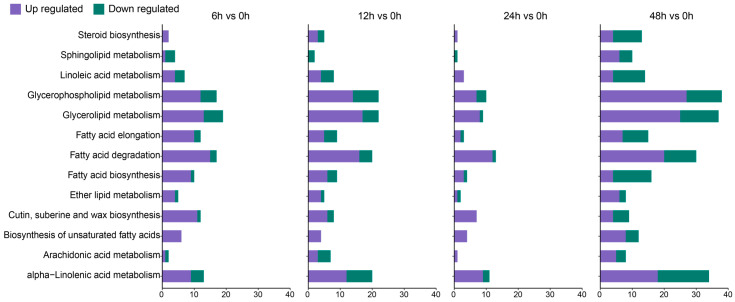
Functional categorization of lipid-associated DEGs modulating multiple metabolic networks of lipid.

A total of 124 lipid-metabolism-related genes were identified in transcriptomic analysis based on GO and KEGG annotations of the comparative group ‘‘T6 h vs. T0 h” (Figure 4), among which a total of 95 were upregulated and 29 were downregulated. Similarly, we retrieved a total of 142 (95 upregulated and 47 downregulated), 69 (58 upregulated and 11 downregulated), and 248 (141 upregulated and 107 downregulated) lipid-related genes in the comparative groups “T12 h vs. T0 h”, “T24 h vs. T0 h”, and “T48 h vs. T0 h”, separately (Figure 4). An upsurge in upregulated genes in the final salt stress stage indicated the activation of many lipid-associated metabolism pathways, with higher gene counts found in the following network categories: glycerophospholipid metabolism, wax biosynthesis, fatty acid degradation, and elongation; however, a marked upsurge in galactolipid-biosynthesis-associated genes was also observed. The expression of DGD1 and DGD2 was significantly upregulated in the comparative groups of 24 h vs. 0 h and 48 h vs. 0 h, which directly reflected a late response of DGD genes towards the salt stress, thus suggesting crucial damage to the plastid membranal structure (Table S1).

The leaf chlorophyll contents significantly decreased with longer exposure to salt stress (Figure 4), and chlorophyll absolutely played a critical role in photosynthesis, light harvesting, and light reactions. Herein, we demonstrated that the expressions of the chlorophyll biosynthetic and metabolic-process-related DEGs were severely suppressed. These included the divinyl chlorophyllide 8-vinyl-reductase activity (DAR) gene, which catalyses chlorophyll biosynthesis, as well as the tetrapyrrole-biosynthesis-related gene, which serves as the primary chlorophyll pigment (Table S1). Based on this evidence, salt stress drastically reduced the substrate contents for chlorophyll biosynthesis, which, in turn, caused yellow leaves.

**Figure 5:**
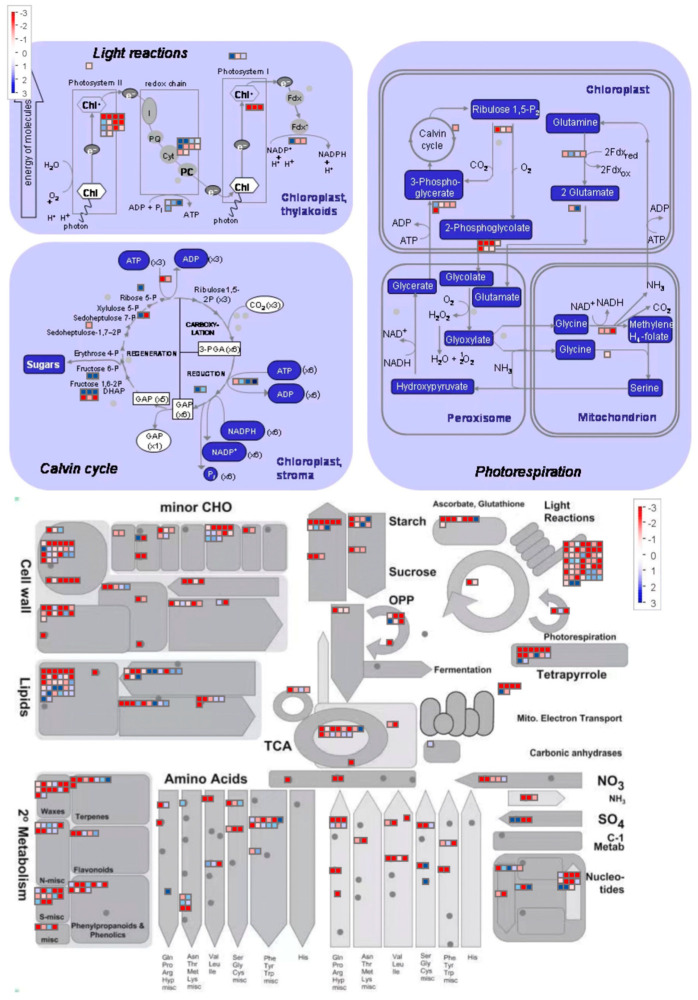
An illustrated schematic overview of associated metabolic pathways.

Meanwhile, the photosynthesis-related DEGs also exhibited a time-dependent response, which was significantly downregulated under salt stress (Figure 5). These included genes in the LHC and Psb families, which serve as light receptors and regulators of excitation energy in photosystems I and II [31]. Similarly, 10 DEGs associated with the PSII reaction centre subunits and plastid thylakoid membrane biosynthesis were identified as downregulated upon salt stress exposure (Table S1). Altogether, this evidence indicates that salt stress rapidly depleted the substrate for chlorophyll biosynthesis with the simultaneous augmentation of chlorophyll degradation; however, in the photosynthetic system, the chlorophyll-interaction-associated genes (PSI and PSII reaction centre subunit) decreased remarkably. Hence, the response of melon to salt injury was to weaken the components of the photosynthetic system.

The root development involves cell wall loosening that is mediated by certain proteins, such as xyloglucan endotransglucosylase/hydrolase (XTH) and peroxidase (POD) [39,40]. During plant morphogenesis, xyloglucan transglucosylase transfers xylglucan into the cell wall, thereby affecting cell wall formation and degradation. Hence, its function is critical to plant secondary development, disease resistance, and stress resistance [41]. As expected, we identified five DEGs coding for XTHs and POD metabolic processes, and the screened genes possessed a similar overall downregulation profile. Salt stress could affect the gene expression trends concerned with auxin (IAA) synthesis and alter its distribution within root tips, which, in turn, results in the retardation of taproot growth and the formation of lateral root primordium while simultaneously enhancing the elongation of existing lateral roots [17,42]. Herein, two auxin homeostasis regulator DEGs, as well as the metabolic process, were strongly suppressed by salt stress (Table S1). This may potentially explain the reduced root growth in the presence of salt stress. Conversely, we identified two elevated genes that modulated IAA synthetase. This may explain the salt-stress-induced alteration in root architecture.

### 3.5. Identification of WGCN-Associated DEGs under Salt Stress

**Figure 6:**
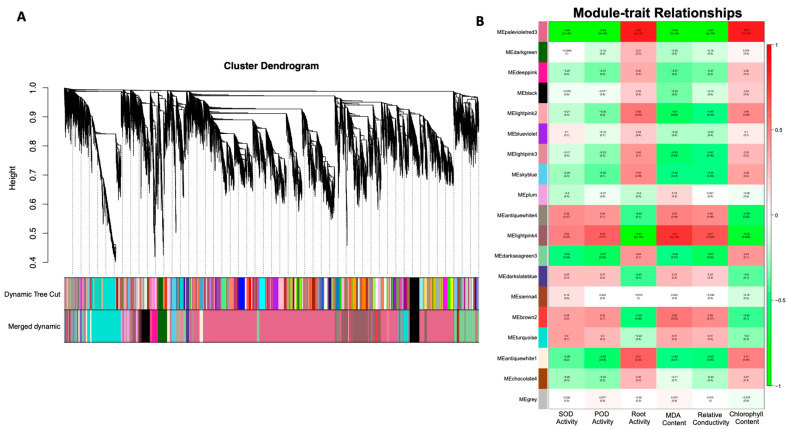
WGCN analysis of identified DEGs in melon variety grown under salt stress treatment. (**A**) Cluster dendrogram exhibiting associated co-expressed gene modules. (**B**) Physiological index module exhibiting the significant correlations and *p*-values, respectively.

The WGCN networks were typically generated based on pair-wise associations between gene expressions of all the samples. Herein, a total of 19 modules were screened by WGCN analysis, as depicted in the visualized dendrogram (Figure 6A), and module–trait association analysis significantly revealed that both MEpalevioletred3 and MElightpink4 network modules were strongly associated with salt stress (Figure 6B). The analysis of GO enrichment terms identified the presence of genes in separate modules and revealed that the depicted membrane (including the integral and intrinsic membrane component), cellular process, and intracellular organelle were strongly associated with the above-mentioned modules.

We found the top 10 hub genes for radiality values using the WGCN analysis (Figure 6). In particular, a total of two genes (*MELO3C006606.2* and *MELO3C017176.2*) were annotated as chlorophyll-related genes, six genes (*MELO3C025475.2*, *MELO3C023596.2*, *MELO3C010708.2*, *MELO3C004214.2*, *MELO3C022113.2*, and *MELO3C015536.2*) were identified as related to the PS II reaction center subunits, and two genes (*MELO3C006053.2* and *MELO3C004491.2*) were associated with plastid thylakoid membrane (Table 1). Overall, we identified that a majority of the hub genes were intricately linked to the photosynthetic PS II apparatus. Thus, we speculated that the salt-stress-induced influence on photosynthesis was primarily concentrated on PS II.

**Figure 7:**
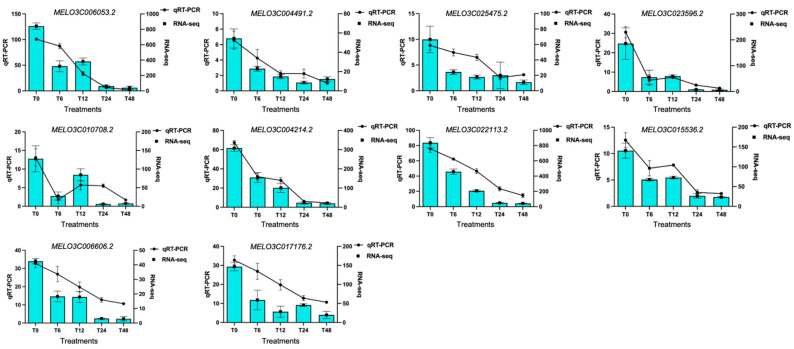
Relative expression profiling of top 10 hub DEGs; line graphs indicate the RNA-seq results and bar graphs exhibit the qRT-PCR results, individually.

So, we performed the experiment of qRT-PCR analysis and further examined the relative expression profiling of hub genes that were identified for various stages of stress. Interestingly, the obtained results were consistent with the expectation that the relative gene expression steadily decreases with the increase in stress (Figure 7). Further, the gene expression profiles at various salt stress stages were comparable to the RNA-seq data, which also proved the positive data accuracy.

## 4. Discussion

### 4.1. Synergistic Network Response of Lipid Metabolism and Photosynthesis in Membrane

Membrane-associated lipids are typically categorized into bilayer and non-bilayer [43]. Many pieces of evidence have suggested that both bilayer and non-bilayer lipids are critical for proper membrane function [44,45]. In particular, the lipid bilayer works for membranal stability, and the non-bilayer mediates protein connectivity and enhances the flexibility of lipid bilayer structures [44]. The lipid bilayer contains PC, PG, PI, DGDG, and SQDG; however, the non-bilayer is made up of PA, PE, PS, and MGDG [45]. Previous studies have demonstrated the crucial significance of MGDG and DGDG in photosynthesis, which could also be easily influenced by abiotic stress [46].

**Figure 8:**
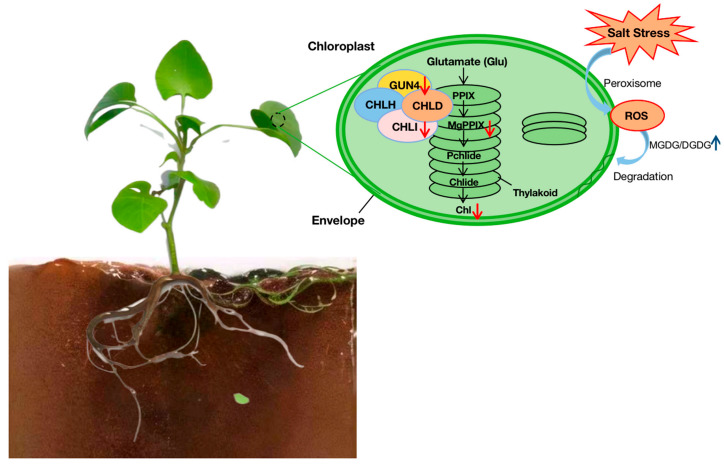
Synergistic network response of membrane–lipid metabolism and photosynthesis function in melon seedling towards salt-induced stress. Blue and red arrows indicate up- and downregulation mechanisms, respectively.

In our study, the DGDG and MGDG contents were reduced, and it was proven that the reduction in glycolipids also caused a reduction in endogenous chlorophyll content in the leaves [47]. This indicated that the photosynthetic membranes suffered severe damage, and the observation of the ultrastructure of chloroplasts also proved that the photosynthetic function of the plant was damaged by salt stress (Figure 8). There is validated evidence that the DGDG-to-MGDG ratio is relatively essential for the stable permeability of photosynthetic membranes [48], and changes within the composition of membrane lipids are a potential attempt to counteract the damage to the photosynthesis-activity-related membrane under stress [15]. For instance, a declined MGDG/DGDG ratio under cold stress helps enhance membranal stability [13,14]. In contrast, the high MGDG-to-DGDG ratio further indicated that salt stress negatively impacted the plastid membranal structure, composition, and uneven photosynthetic membranal homeostasis.

Our current study exhibited that the chlorophyll content in melon leaves decreased drastically with prolonged salt stress treatment, consistent with the reduced chlorophyll concentration described in other crop plants under salt stress [49,50]. As the most important plant pigment, chlorophyll also serves as an indicator for enriched physiological conditions, photosynthetic efficacy, and cellular metabolism [51,52,53]. However, the decreased chlorophyllase activity and irregular expression arrays of chlorophyll biosynthesis and degradation-related genes caused excessive ROS and severely reduced plant chlorophyll levels [54]. The tetrapyrrole biosynthesis pathway (TBP) is a significant process for chlorophyll production, and 5-aminolevulinic acid (ALA) synthesis and magnesium chelase (MgCh) are two essential nodes that regulate chlorophyll production [55]. MgCh is a multi-subunit complex made up of CHLH, CHLI, and CHLD, and it contributes to the first step of chlorophyll synthesis branch reaction [56]. GUN4 is a common protein present within oxygen-releasing photosynthetic organisms that associates with the MgCh catalytic subunit CHLH and interacts with its substrate and products to stimulate activity as well as modulate chlorophyll production [57], and any genetic changes can negatively impact chlorophyll synthesis during these processes. In the present study, genes associated with the tetrapyrrole-interacting protein and Mg-protoporphyrin IX chelatase were all downregulated by salt stress, which may have triggered the slowdown of chlorophyll synthesis and ultimately diminished chlorophyll content.

On the other hand, chloroplasts primarily act as the major source of photosynthesis and ROS production and are very sensitive to salt-induced stress [58]. When this stress affects the chloroplast, the chloroplast structure and function become damaged, and the production and accumulation activity of ROS become unregulated [59,60]. Our study results (Figure 2A–E) also exhibited that the melon photosynthetic-associated organelle structure was severely damaged with the extended time period of salt stress at 48 h, which showed stunted growth and leaf-yellowing symptoms. According to our WGCN analysis, most of the hub genes were related to PSII, which significantly indicated that the PSII reaction center was the primary region affected by salt-stress-induced oxidative damage. These results are consistent with Arena et al. [61], who checked the effects of cadmium and lead stress on plant uptake by roots and shoots, changes in cell ultrastructure and photosynthetic efficiency, and photosynthetic key protein levels. The obtained results demonstrate why the PSII quantum yield of linear electron transport, the electron transport rate, and the maximal PSII photochemical efficiency are often used as plant stress indicators to describe the capability of a photosynthetic apparatus to utilize absorbed light in photochemical reactions.

### 4.2. Enzymes, Non-Enzymatic Factors, and Hormones Inducing Retarded Root Growth

Crucial damage to the cell membrane is the initial process that appears in stress-affected plants. Further, the integrity and stability of the cell membrane are intricately linked to stress tolerance exposure [62], and excessive ROS induced by lipid peroxidation will reduce membrane integrity [63]. Furthermore, the accumulating ROS enhances oxidative damage, thereby diminishing plant growth and root development [64]. Herein, we used MDA content and electrolyte leakage as indicators for observing the damage to the membrane. In addition, the marked upsurge in the above-mentioned indices in this study indicated severe damage to cell membranes under salt stress.

Root development is highly dependent on enzymes that modulate cell wall biosynthesis and metabolic processes, for example, xyloglucan endotransglucosylase/hydrolase (XTH) and POD [39,40]. During plant morphogenesis, xyloglucan transglucosylase transfers xylglucan into the cell wall, thereby affecting cell wall formation and degradation. Hence, its function is critical to plant secondary development, disease resistance, and stress resistance [41]. Plant cell development and morphogenesis are primarily regulated by the dynamic plant primary cell wall structure, which, in turn, is maintained by both non-enzymatic and enzymatic factors [65]. Among the various remodelling enzymes of the cell wall, the xyloglucan endotransglucosylase/hydrolase (XTH/XET) gene family produces enzymatic proteins that enhance cell expansion and division. Thus, these proteins may potentially respond to unfavourable stress conditions. Herein, we identified seven XTHs as salt-stress-induced DEGs, and a majority of these gene expressions were drastically suppressed in the later stage of stress.

Salt stress can induce cell wall stiffening, a process that likely involves peroxidase activity. Moreover, a potential link between POD and cell expansion restriction has been reported in multiple earlier publications [66,67]. The lower POD expressions may augment oxygen radical accumulation, which, in turn, can damage membrane integrity, thereby affecting relative fatty acid quantity, which may result in cell collapse and stunted root growth. Herein, we provide potential physiological reasons behind the salt-stress-related retardation of plant growth. The previous investigations similarly revealed a strong correlation between plant IAA signalling and salt stress [68]. Salinity severely impacts IAA homeostasis through an alteration of IAA metabolism [69], and our results also validated this finding, as multiple IAA biosynthesis- and homeostasis-related genes were differentially synchronized by salt stress. Furthermore, it has been demonstrated that IAA also serves as a typical integrator for numerous internal (physical) and external (environmental) stimuli that modulate lateral root development [70]. This suggests that in our experimental study, the salt-stress-induced stunted lateral root growth was caused by physiological variations in signal transduction and IAA pathways.

## 5. Conclusions

Salinity negatively modulates the development of roots and photosynthetic responses in plants. The leaves and roots are indispensable regions that are most sensitive to salt-induced stress and primarily respond to it. Hence, severely elevated MDA and REC levels, along with ROS accumulation and reduced chlorophyll and root activity brought on by a superoxidized membranal system, can completely explain the abnormal development of plants under stress. The results of our salt-induced treatments and analysis of physiological indices showed that the salt-induced injury affected the growth and development of the melon plant. The symptoms of leaf yellowing were caused by changes in the leaf’s photosynthetic activity and lipid membrane composition, and these changes caused much less effective photosynthesis as well as an irregular internal structure of chloroplasts. In addition, our in-depth molecular analysis revealed that the PSII reaction centre may be the primary stress target because we identified strong suppression of the cell wall biosynthesis- and IAA-metabolism-related genes under salt stress treatment, which, in turn, severely decreased the root activity and retarded root growth.

## Figures and Tables

**Table 1:** Significant annotation of top ten hub genes.

Gene ID	Gene Annotation
*MELO3C006053.2*	Protein CURVATURE THYLAKOID 1A, chloroplastic
*MELO3C004491.2*	Thylakoid lumenal 16.5 kDa protein, chloroplastic
*MELO3C025475.2*	psbP-like protein 1, chloroplastic
*MELO3C023596.2*	Photosystem II stability/assembly factor HCF136, chloroplastic
*MELO3C010708.2*	photosystem II core complex proteins psbY, chloroplastic
*MELO3C004214.2*	Chlorophyll a-b binding protein, chloroplastic
*MELO3C022113.2*	PS II-associated light-harvesting complex II
*MELO3C015536.2*	photosystem II D2 protein-like
*MELO3C006606.2*	Divinyl chlorophyllide a 8-vinyl-reductase, chloroplastic
*MELO3C017176.2*	chlorophyll biosynthetic process

## Data Availability

The original contributions presented in the study are included in the article and Supplementary Material. The RNA-seq data have been uploaded to NCBI (accession number: PRJNA987131) and additional queries can be directed at the corresponding author.

## Supplementary Materials

The relevant additional dataset information can be downloaded at: https://www.mdpi.com/article/10.3390/genes14091728/s1, Figure S1: Evaluation of gene expression profiles among 15 samples under salt stress. (A) Venn diagram of DEG distributions, (B) DEG quantities in each comparison, (C) Heatmap of correlation coefficients of 5 DEG samples (T0-T48); Figure S2: Gene ontology categorization of DEGs. DEGs were categorized based on gene ontology (GO) annotation. The proportion of each category is displayed based on the ontology-based biological process, cellular component, and molecular function; Figure S3: Top 20 KEGG pathway enrichment of DEGs that were (A) upregulated following salt stress for 6 h, (B) upregulated following salt stress for 12 h, (C) upregulated following salt stress for 24 h, and (D) upregulated following salt stress for 48 h, (E) downregulated following salt stress for 6 h, (F) downregulated following salt stress for 12 h, (G) downregulated following salt stress for 24 h, and (H) downregulated following salt stress for 48 h; Figure S4: Significant GO terms enriched in the DEGs of the two network modules. (A) the top 30 enriched terms in MElightpink3, (B) the top 30 enriched terms in MElightpink4; Table S1: DEGs related to key biological processes.
