# Supplementary material for: RNA-Seq Identified Putative Genes Conferring Photosynthesis and Root Development of Melon under Salt Stress
Source: Genes (Basel). 2023 Aug 29;14(9):1728. doi: 10.3390/genes14091728 (PMC10530605; doi:10.3390/genes14091728)
Supplement: Supplementary file 1 [file genes-14-01728-s001.zip › genes-2555067-supplementary.pdf]

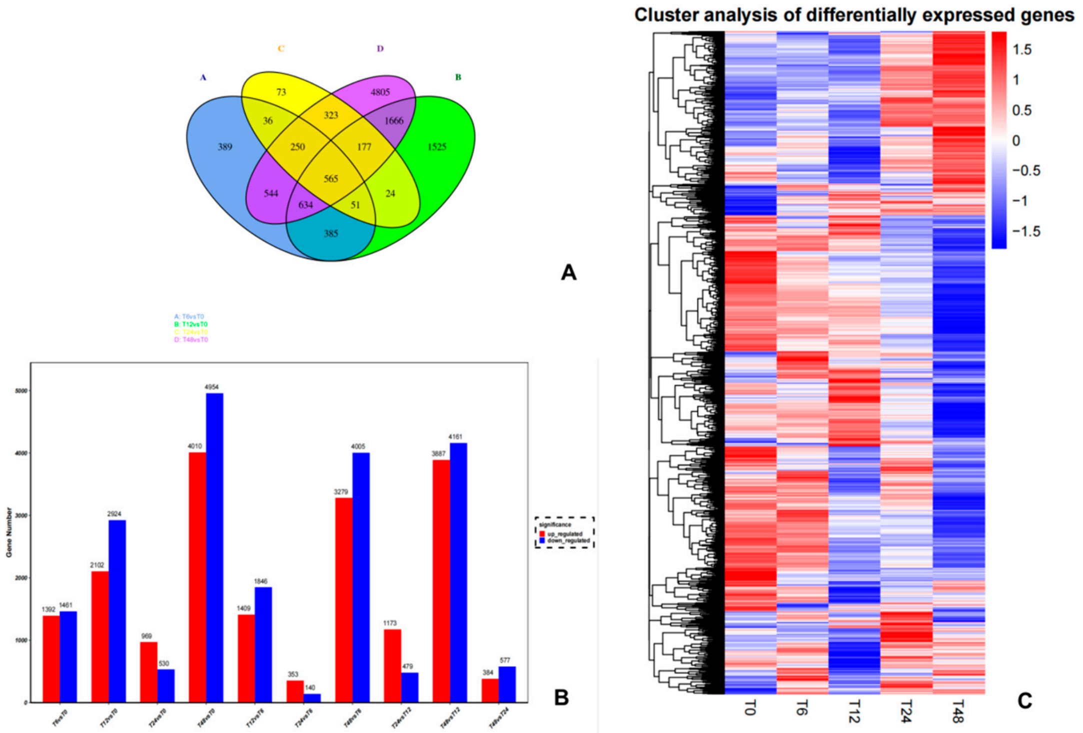

Figure S1. Evaluation of gene expression profiles among 15 samples under salt stress. (A) Venn diagram of DEG distributions. (B) DEG quantities in every comparison. (C) Heatmap of correlation coefficients of 5 DEG samples (T0-T48).

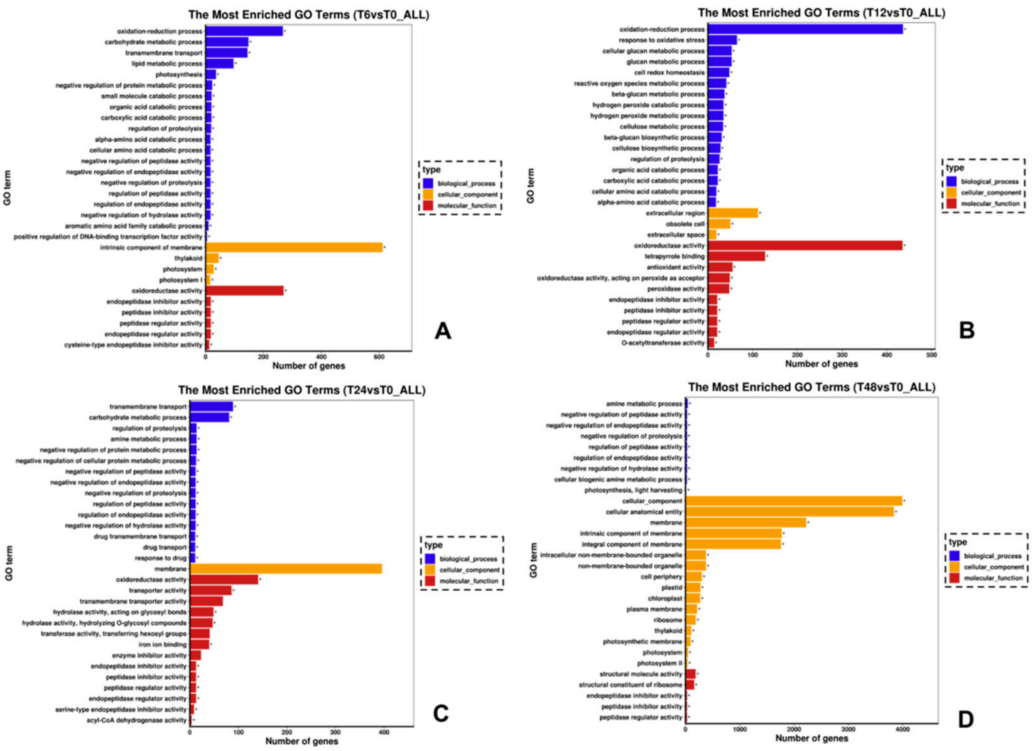

Figure S2. Gene ontology categorization of DEGs. DEGs were categorized based on gene ontology (GO) annotation. The proportion of each category is displayed based on the ontology-based biological process, cellular component, and molecular function.

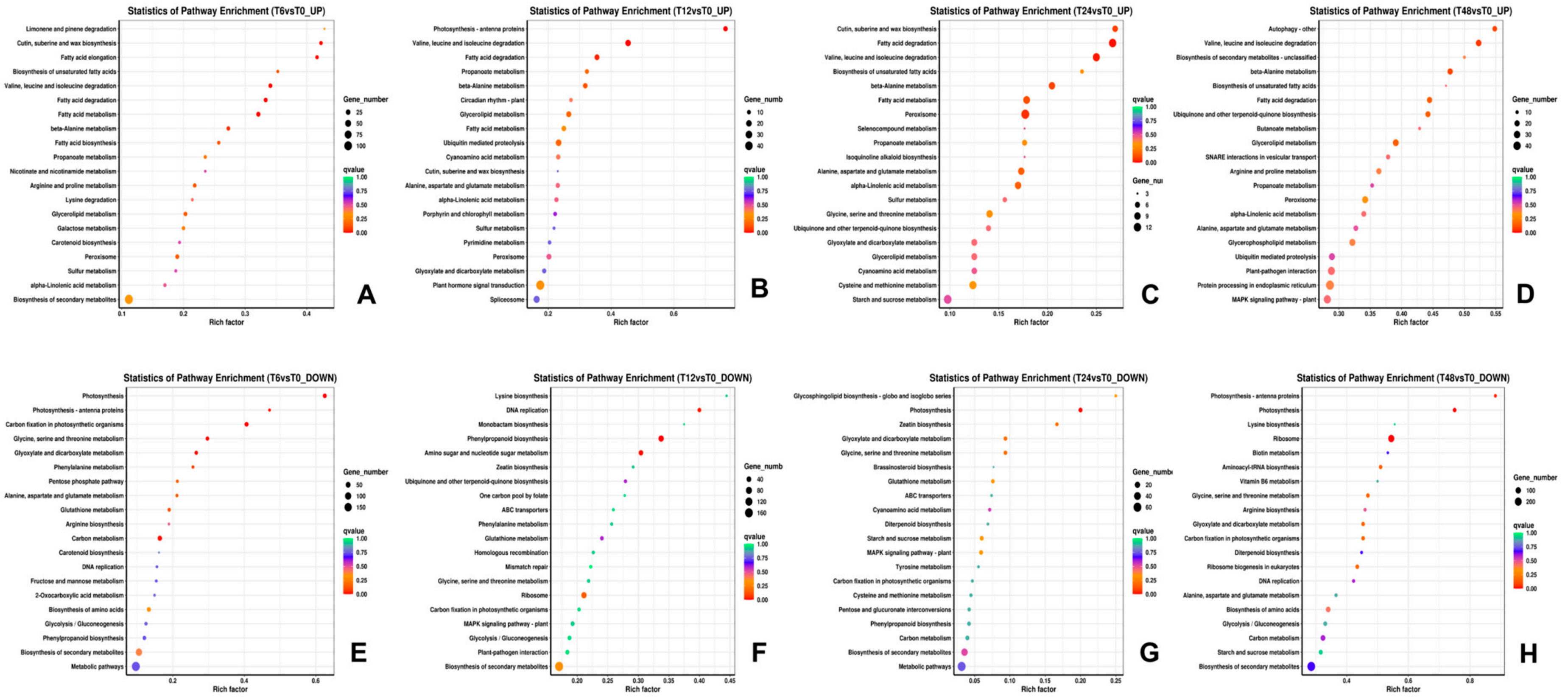

Figure S3. Top 20 KEGG pathway enrichment of DEGs that were (A) upregulated following salt stress for 6 h, (B) upregulated following salt stress for 12 h, (C) upregulated following salt stress for 24 h, and (D) upregulated following salt stress for 48 h. (E) downregulated following salt stress for 6 h, (F) downregulated following salt stress for 12 h, (G) downregulated following salt stress for 24 h, and (H) downregulated following salt stress for 48 h.

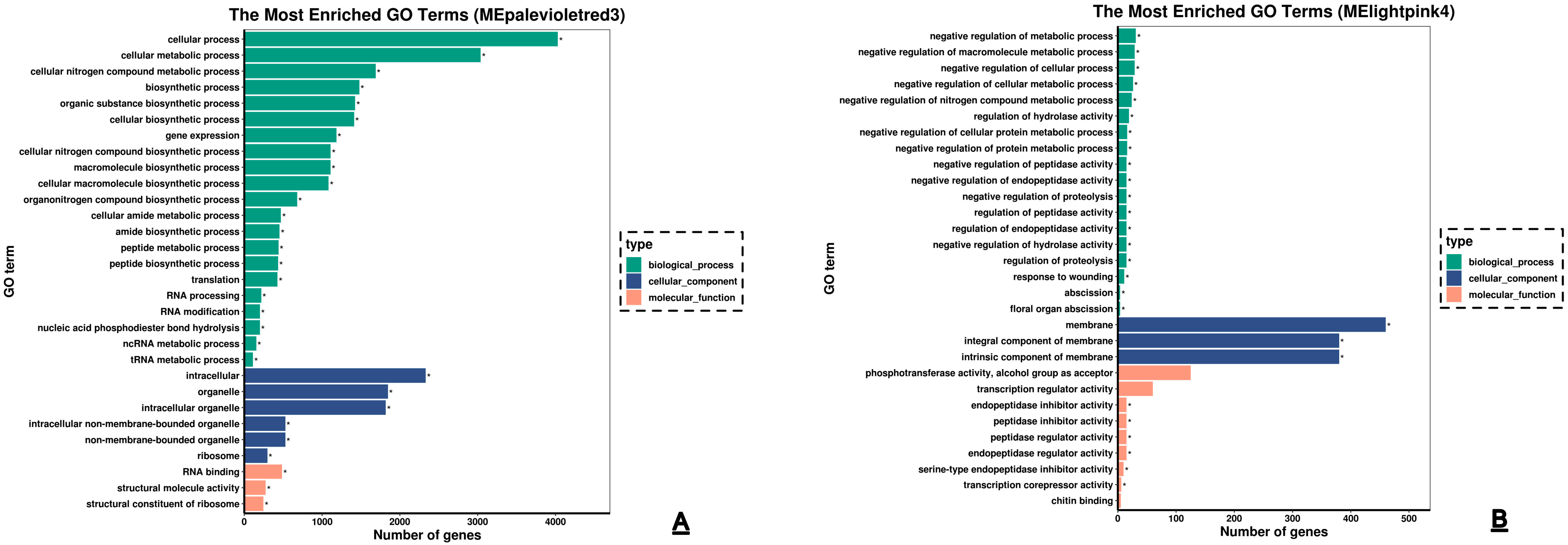

Figure S4. Significant GO terms enriched in the DEGs of the two network modules. (A) the top 30 enriched terms in MElightpink3, (B) the top 30 enriched terms in MElightpink4

Table S1. DEGs related to key biological processes

Functional annotation	Gene ID	FPKM value				
		T0	T6	T12	T24	T48
glycerolipid metabolic process(DGD1)	*MELO3C009290.2*	34.19	23.24	15.74	32.98	58.16
glycerolipid metabolic process(DGD2)	*MELO3C003088.2*	7.99	17.92	13.01	34.29	61.62
photosynthesis, light harvesting in photosystem I (LHC)	*MELO3C022113.2*	436.14	190.47	1083.48	175.24	62.58
chlorophyll A-B binding protein(LHC)	*MELO3C003238.2*	559.47	146.71	142.22	269.58	169.65
photosynthesis, light harvesting (LHC)	*MELO3C015536.2*	140.70	65.58	80.16	46.34	23.44
chlorophyll A-B binding protein(LHC)	*MELO3C016556.2*	125.66	60.25	56.40	67.84	3.55
photosystem II assembly (PsbP)	*MELO3C025475.2*	66.45	24.30	17.76	19.94	11.00
photosystem II reaction center W protein(PsbW)	*MELO3C004388.2*	102.40	37.47	12.94	24.95	10.67
Photosystem II stability, chloroplastic(PsbD)	*MELO3C023596.2*	185.51	55.27	59.37	54.51	6.66
photosystem II assembly (PsbP)	*MELO3C025475.2*	66.45	24.30	17.76	19.94	11.00
plastid thylakoid membrane(PsbS)	*MELO3C015536.2*	140.70	65.58	80.16	46.34	23.44
Thylakoid lumenal 16.5 kDa protein, chloroplastic	*MELO3C004491.2*	54.35	22.93	14.90	15.33	12.21
NAD(P)-binding Rossmann-fold superfamily protein	*MELO3C024358.2*	43.11	14.69	5.71	8.15	1.01
chlorophyll biosynthetic process (DAR)	*MELO3C006606.2*	42.44	18.29	17.92	13.11	2.94
Chlorophyllase	*MELO3C002961.2*	1.25	1.03	0.00	0.94	0.51
Mg-protoporphyrin IX monomethyl ester cyclase	*MELO3C026802.2*	77.99	34.18	54.06	51.01	36.73
Mg-protoporphyrin IX chelatase(CHLI)	*MELO3C007233.2*	178.38	77.70	53.54	60.27	18.14
Tetrapyrrole-binding protein, chloroplastic(GUN4)	*MELO3C011657.2*	71.29	22.94	31.18	18.35	5.46
xyloglucan endotransglucosylase/hydrolase	*MELO3C017476.2*	1.68	0.38	0.09	1.24	0.07
xyloglucan endotransglucosylase/hydrolase	*MELO3C017476.2*	1.24	0.17	0.24	1.03	0.10
xyloglucan endotransglucosylase/hydrolase	*MELO3C018033.2*	1.68	0.38	0.09	1.24	0.07
Peroxidase	*MELO3C002457.2*	672.03	99.27	25.89	201.90	1.35

(next page)

(continued)

Peroxidase	*MELO3C010627.2*	83.20	33.68	30.83	37.67	28.36
Auxin biosynthetic process	*MELO3C007877.2*	0.55	1.35	0.64	0.39	0.16
Protein LATERAL ROOT PRIMORDIUM 1	*MELO3C015123.2*	9.51	2.19	1.90	1.93	0.67
Indole-3-acetic acid-amido synthetase GH3.3	*MELO3C027346.2*	3.30	20.22	2.71	90.24	112.88
Indole-3-acetic acid-amido synthetase GH3.3	*MELO3C007597.2*	1.54	7.47	1.71	76.76	46.53
